# The Benefit of Hydrogen Gas as an Adjunctive Therapy for Chronic Obstructive Pulmonary Disease

**DOI:** 10.3390/medicina60020245

**Published:** 2024-01-30

**Authors:** Shih-Feng Liu, Chin-Ling Li, Hui-Ching Lee, Hui-Chuan Chang, Jui-Fang Liu, Ho-Chang Kuo

**Affiliations:** 1 Department of Respiratory Therapy, Kaohsiung Chang Gung Memorial Hospital, Kaohsiung 833, Taiwan; liuphysico@yahoo.com.tw (S.-F.L.); musquito16@cgmh.org.tw (C.-L.L.); katherinelee8025440@yahoo.com.tw (H.-C.L.); elaine11142@cgmh.org.tw (H.-C.C.); 2 Division of Pulmonary and Critical Care Medicine, Department of Internal Medicine, Kaohsiung Chang Gung Memorial Hospital, Kaohsiung 833, Taiwan; 3 College of Medicine, Chang Gung University, Taoyuan 333, Taiwan; 4 Department of Respiratory Care, Chang Gung University of Science and Technology, Chiayi 600, Taiwan; chin9688@yahoo.com.tw; 5 Chronic Diseases and Health Promotion Research Center, Chang Gung University of Science and Technology, Chiayi 600, Taiwan; 6 Department of Paediatrics, Kaohsiung Chang Gung Memorial Hospital, Kaohsiung 833, Taiwan

**Keywords:** chronic obstructive pulmonary disease, hydrogen gas adjuvant therapy, therapeutic benefit

## Abstract

*Background and Objectives:* Recent studies suggest that hydrogen gas possesses anti-inflammatory, antioxidant, and anti-apoptotic properties. This study aimed to explore the therapeutic potential of hydrogen gas and assess its safety and tolerability in individuals with chronic obstructive pulmonary disease (COPD). *Materials and Methods:* Enrolled COPD patients received standard treatments along with additional hydrogen inhalation for 30 min in the morning, afternoon, and evening over a 30-day period. The assessment included changes in the COPD Assessment Test (CAT), the modified Medical Research Council (mMRC) Dyspnea Scale, lung function, sleep quality, inflammation markers, and oxidative stress markers before and after hydrogen inhalation. *Results:* Six patients participated in this study. Patients 2, 3, 4, 5, and 6 demonstrated improvements in CAT scores following hydrogen gas intervention, with patients 2, 4, 5, and 6 also showing improvements in mMRC scores. Statistically, this study revealed significant improvements in CAT [15.5 (10.5–19.75) vs. 8.5 (3–13.5); *p* = 0.043] and mMRC scores [2.5 (1–4) vs. 2 (0–3.25); *p* = 0.046] before and after intervention, respectively. However, no significant differences were observed in lung function, DLCO, sleep quality, and 6 MWT before and after hydrogen therapy. CBC examination showed a significant difference in platelet count before and after treatment [247 (209.75–298.75) vs. 260 (232.75–314.5); *p* = 0.043], respectively, while other blood tests, inflammation markers, and oxidative stress markers did not exhibit significant differences before and after hydrogen therapy. All patients experienced no obvious side-effects. *Conclusions:* Adjuvant therapy with hydrogen gas demonstrated symptom improvements in specific COPD patients, and no significant adverse effects were observed in any of the patients. Hydrogen gas may also exert a modulatory effect on platelet count.

## 1. Introduction

Chronic obstructive pulmonary disease (COPD) is a progressive and debilitating respiratory ailment characterized by chronic bronchitis and emphysema, leading to breathing difficulties and impaired lung function [1]. As a significant global health concern, COPD affects millions of individuals worldwide, resulting in substantial morbidity and mortality. The pathogenesis of COPD involves oxidative stress and inflammation, and recent research has shed light on the potential benefits of hydrogen gas. Known for its antioxidant and anti-inflammatory properties, hydrogen gas has shown promise in alleviating COPD in both preclinical and clinical settings [2,3,4].

The anti-inflammatory and antioxidant effects of hydrogen gas are attributed to its ability to target reactive oxygen species (ROS) and inhibit NLRP3 inflammasome activation in macrophages [5]. Furthermore, hydrogen gas plays a role in regulating the expression of genes associated with oxidative stress and inflammation, including heme oxygenase-1 and nuclear factor-kappa B (NF-κB) [6,7]. It also modulates the levels of inflammatory cytokines and chemokines, such as interleukin-1 beta (IL-1β), interleukin-6 (IL-6), and tumor necrosis factor alpha (TNF-α), by inhibiting the activation of the transcription factor NF-κB [8]. Additionally, hydrogen gas regulates the expression of antioxidant enzymes like superoxide dismutase (SOD) and catalase, providing protection against oxidative stress-induced damage [9].

Current COPD therapies primarily focus on symptom reduction, improved lung function, and prevention of exacerbations. However, there is a growing interest in exploring novel therapeutic interventions to further alleviate symptoms, slow disease progression, and enhance patients’ quality of life. One such promising avenue is the investigation of hydrogen gas as a potential treatment for COPD. This emerging research holds the potential to open new doors in the development of innovative approaches to manage COPD, offering hope for improved outcomes and a better quality of life for affected individuals.

## 2. Materials and Methods

### 2.1. Study Design

Between 1 July 2021 and 30 June 2023, we selected COPD patients in the outpatient department at Kaohsiung Chang Gung Hospital who expressed willingness to participate in this research project. These patients had previously received standard treatment according to the GOLD guidelines (i.e., SABA/LABA medication therapy, corticosteroids/other medications, or long-term oxygen therapy) with stable conditions. After inclusion, they began receiving 30 days of hydrogen inhalation therapy at home. This study aimed to investigate the synergistic effects and assess the safety and tolerability of adjunctive inhaled hydrogen in COPD. Assessment parameters included changes in the COPD Assessment Test (CAT), modified Medical Research Council (mMRC) Dyspnea Scale, lung function, sleep quality, inflammation markers, and free radicals before and after a 30-day treatment period.

A. Inclusion criteria (all conditions must be met):

1. Patients aged ≥ 40 years with written consent.

2. Diagnosis of COPD based on the GOLD guidelines.

Diagnosis criteria include a fixed ratio of FEV1/FVC less than 0.7 after bronchodilator inhalation, indicating persistent airflow limitation. The severity of airflow limitation is classified according to GOLD criteria^1^:

GOLD 1 (mild): FEV1 ≥ 80% of predicted value.

GOLD 2 (moderate): 50% ≤ FEV1 < 80% of predicted value.

GOLD 3 (severe): 30% ≤ FEV1 < 50% of predicted value.

GOLD 4 (very severe): FEV1 < 30% of predicted value.

B. Exclusion criteria:

1. Exclusion based on chest X-ray and medical history to rule out other causes of airflow limitation, such as pulmonary tuberculosis, bronchial asthma, bronchiectasis, and congestive heart failure.

2. Patients unable to comply with the study requirements or clinical assessments.

3. Patients not meeting the inclusion criteria as determined by the principal investigator.

### 2.2. Adjunctive Hydrogen Inhalation

Patients undergoing adjunctive hydrogen inhalation would receive treatment using a hydrogen–oxygen machine for 30 min each in the morning, afternoon, and evening throughout the treatment period at home. The patients would inhale a gas mixture containing 73% hydrogen and 27% oxygen through a nasal tube, with the gas flow rate set at 70–75 L per hour. In the event of any abnormal respiratory, heart rate, blood oxygen, or blood pressure readings occurring during the use of the hydrogen–oxygen machine, or if the patient is unable to cooperate, or if the trial needs to be terminated for any reason at the subject’s request, prompt cessation of the treatment would be carried out.

### 2.3. Instrumentation and Interpretation of Possible Side-Effects

In this study, we employed the EIOXY Machine HB-133 model, provided by EPOCH ENERGY TECHNOLOGY CORP (Kaohsiung, Taiwan). The machine operates on AC power supply (110 V, 50/60 Hz), with a maximum power usage of 0.35 kW. Operating at a non-accumulated working pressure of 0 kg/cm^2^, the machine has maximum water consumption per cycle of 25 ± 5 c.c. Its weight, excluding accessories, is 38.5 kg, and its dimensions measure 360,350,620 mm. A reserved space of 100 mm (front, back, left, right, and top) is necessary. The machine produces a gas flow of 70–75 L/H, consisting of 73% hydrogen and 27% oxygen, sufficient for normal human respiration. Extensive research and numerous papers have consistently confirmed that this combination of hydrogen and appropriate oxygen results in zero side-effects. The primary consideration is to prevent nasal dryness when using a nasal tube with inhalation devices In exceptionally rare cases, nasal skin abrasion may lead to minor nosebleeds. However, this issue can be effectively addressed by adjusting the nasal tube length to minimize the occurrence.

### 2.4. Clinical and Laboratory Variables

Clinical and laboratory variables were assessed before and after 30 days of adjunctive hydrogen inhalation, encompassing the following parameters: CAT and mMRC scores; sleep quality; pulmonary function test; Diffusing capacity of the lung for carbon monoxide (DLCO); 6 min walk test (6 MWT); complete blood cell count and differential count; oxidative stress markers (plasma glutathione peroxidase (GPX), myeloperoxidase (MPO), total anti-oxidant capacity (TAC), urine 8-hydroxy-2′-deoxyguanosine (8-OHdG)); lipid profiles (T-CHO, HDL, LDL, TG).

### 2.5. Statistical Analysis

Continuous variables are presented as median, Interquartile Range (IQR). All objective and subjective data were recorded before and after the use of inhaled hydrogen adjuvant therapy. The Wilcoxon signed-rank test was employed to assess pre- and post-treatment lung function, 6 MWD, CAT, mMRC, Borg scale, inflammation markers, and free radicals. Calculations were conducted using SPSS 26, with a defined *p*-value of less than 0.05 indicating significant differences.

## 3. Results

The basic characteristics of the six patients with COPD receiving inhaled hydrogen adjuvant therapy are presented in Table 1. This study included six participants, comprising five males and one female, with an average age of 68 years. Five participants had a smoking history ranging from 15 to 45 pack-years, while one participant had never smoked. The post-bronchodilator FEV1/FVC had an average of 49.8%. The first patient had a GOLD severity classified as severe, while the other five patients had moderate severity (Table 1).

**Figure 1:**
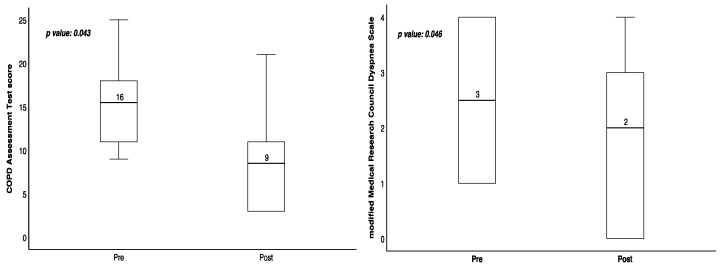
CAT and mMRC of 6 patients with chronic obstructive pulmonary disease before and after inhaled hydrogen adjuvant therapy intervention.

**Figure 2:**
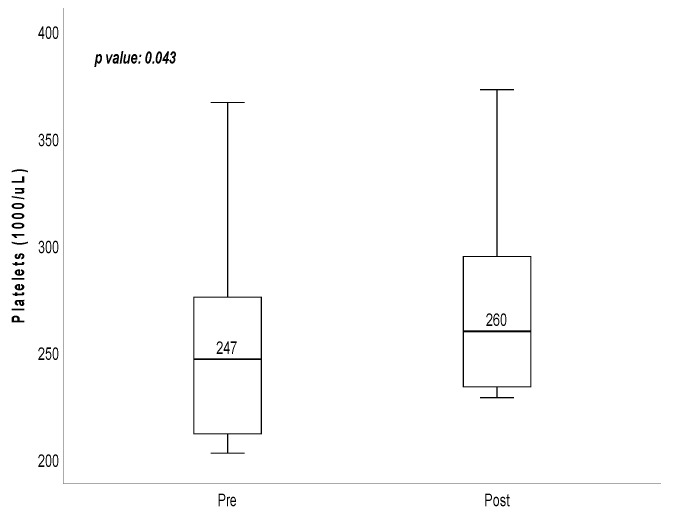
Platelets of 6 patients with chronic obstructive pulmonary disease before and after inhaled hydrogen adjuvant therapy intervention.

Patients 2, 3, 4, 5, and 6 demonstrated improvements in CAT scores following hydrogen gas inhalation intervention, and patients 2, 4, 5, and 6 also exhibited improvements in mMRC scores after the intervention (Table 1). Statistically, this study demonstrated significant improvements in CAT [15.5 (10.5–19.75) vs. 8.5 (3–13.5); *p* = 0.043] and mMRC scores [2.5 (1–4) vs. 2 (0–3.25); *p* = 0.046] before and after hydrogen gas inhalation, respectively (Table 2, Figure 1). However, no significant differences were observed in lung function, DLCO, sleep quality, and 6 MWT before and after hydrogen therapy (Table 1). CBC examination revealed a significant difference in platelet count before and after treatment [247 (209.75–298.75) vs. 260 (232.75–314.5); *p* = 0.043] (Table 3, Figure 2), respectively, while other blood tests, inflammation markers, and oxidative stress markers did not show significant differences before and after hydrogen therapy (Table 3).

Regarding the side-effects of hydrogen therapy, two patients reported dizziness, which subsided with continued usage, and no severe adverse effects were observed. Patient 1, enrolled on 27 December 2021, and commencing hydrogen therapy on December 29, discontinued treatment ahead of schedule on 27 January 2022, due to feeling unwell after receiving a COVID-19 vaccine during the study period. The remaining five patients completed this study as planned.

## 4. Discussion

In our study, the use of hydrogen gas as adjuvant therapy showed symptom improvement in specific COPD patients (patients 2, 4, 5, and 6) and all patients exhibited no significant adverse effects in both clinical assessments and blood tests before and after the intervention. However, further data collection and analysis are required to pinpoint which subset of the COPD population might derive the most benefit from this therapy. Regarding sleep quality, inflammation markers, oxidative stress markers, and lung function, no significant differences were observed during the one-month usage period. Exploring longer treatment durations or early intervention in COPD patients may yield different outcomes and necessitate additional investigation.

Preclinical studies have indicated that hydrogen gas can mitigate oxidative stress and inflammation in animal models of COPD [2,3,10]. Additionally, hydrogen-rich saline has demonstrated protective effects against acute lung injuries induced by burns or oleic acid in animal models [11,12,13]. The protective attributes of hydrogen are associated with its capacity to reduce oxidative stress, inflammation, and apoptosis [14]. For instance, in a rat model of COPD induced by cigarette smoke exposure, hydrogen-rich saline was shown to diminish airway inflammation and oxidative stress while improving lung function [15]. Similarly, in a mouse model of COPD induced by chronic exposure to cigarette smoke, hydrogen gas inhalation reduced airway inflammation and enhanced lung function, potentially through the inhibition of inflammatory cytokines and oxidative stress markers [16].

In a pilot study, the inhalation of hydrogen gas demonstrated a reduction in airway inflammation and oxidative stress among COPD patients [4]. Wang et al. reported that hydrogen gas improved lung function in a mouse model of COPD by inhibiting inflammation and oxidative stress [2], while Liang et al. found that hydrogen-rich saline improved lung function in a mouse model of COPD by reducing oxidative stress and inflammation [3]. Another clinical study showed that individuals with COPD who received hydrogen-rich saline treatment for 12 weeks experienced improvements in lung function, exercise tolerance, and quality of life [17].

To the best of the authors’ knowledge, the specific effects of hydrogen therapy on dyspnea in COPD patients are not conclusively established, and research in this area is still evolving. Hydrogen therapy is being explored for its potential anti-inflammatory and antioxidant properties, and while there is some evidence suggesting benefits in various conditions, more research is needed to understand its mechanisms and effects in specific respiratory conditions like COPD.

Potential reasons for improvements in dyspnea following hydrogen therapy might include the following. First, anti-inflammatory effects: Hydrogen has been suggested to have anti-inflammatory properties, and inflammation is a key component of respiratory diseases like COPD. Reducing inflammation in the airways may contribute to improvements in breathing and reduced dyspnea. Second, antioxidant effects: Hydrogen is known to have antioxidant effects, which means it can neutralize harmful reactive oxygen species. COPD is associated with oxidative stress, and by reducing oxidative damage, hydrogen therapy might contribute to improved respiratory function. Third, protection against tissue damage: Hydrogen’s potential to protect against tissue damage could be beneficial for preserving lung function. In COPD, structural changes and damage to lung tissues contribute to airflow limitations, and any therapy that helps mitigate this damage may improve symptoms. Nevertheless, our laboratory data, encompassing inflammation markers, oxidative stress markers, and lung function, did not correlate with the symptomatic scores.

Platelet count is a crucial parameter for assessing blood clotting and overall health. Deviations in platelet count can lead to various health issues, including bleeding disorders and thrombosis. Given the increasing attention to hydrogen therapy for its potential health benefits, particularly its antioxidative and anti-inflammatory effects, its potential influence on platelet count is of interest. Research suggests that hydrogen gas might have a modulatory effect on platelet count, although the precise mechanisms are not yet fully understood. Oxidative stress and inflammation, targeted by hydrogen therapy, can impact platelet production and function. By reducing oxidative stress and inflammation, hydrogen therapy might indirectly contribute to maintaining a healthy platelet count. Several studies have explored the potential effects of hydrogen therapy on platelet count [18]. Ohsawa et al. demonstrated that hydrogen-rich water had a preventive effect on an animal model of cerebral infarction, suggesting a potential role in platelet-related events [18]. Additionally, Qian et al. found that hydrogen-rich saline may inhibit collagen-induced platelet aggregation in healthy volunteers’ blood samples. [19]. Takeuchi et al. also demonstrated that hydrogen may inhibit collagen-induced platelet aggregation in both ex vivo and in vivo studies [20]. However, while these initial observations are promising, more extensive research is necessary to establish a clear connection between hydrogen therapy and the modulation of platelet count.

To the best of the authors’ knowledge, the optimal frequency of hydrogen gas administration for COPD remains an area of ongoing research. Hydrogen therapy is a relatively new and developing field, and there is still much to learn about its precise mechanisms, optimal dosages, and frequency of administration for various medical conditions. In our research, the hydrogen therapy for three times a day is just to accommodate the patients’ daily schedules and does not have a genuine theoretical basis.

Elastin breakdown and abnormal elastin deposition have been implicated in the pathogenesis of COPD [21,22]. The degradation of elastin leads to loss of lung elasticity and contributes to the development of emphysema, a common feature of COPD [23]. Desmosine and isodesmosine are often used as biomarkers for elastin degradation. Increased levels of these amino acids in urine or blood samples may indicate increased elastin turnover and could be associated with conditions such as COPD. Hydrogen gas therapy has been investigated for its potential to attenuate inflammation, oxidative stress, and tissue damage in the lungs. By reducing oxidative stress and inflammation, hydrogen therapy may help protect lung tissue and potentially slow down the progression of COPD. Desmosine and isodesmosine may be useful biomarkers of hydrogen gas therapeutic effects in COPD as future research directions.

Additionally, exploring potential synergistic effects of hydrogen therapy with current standard treatments could enhance overall therapeutic outcomes in COPD management. Long-term studies are necessary to evaluate the sustained effects of hydrogen therapy and its potential to modify disease progression in COPD. Identifying specific patient populations more likely to benefit from hydrogen therapy can help personalize treatment approaches in COPD.

Several limitations were present in our study. Currently, hydrogen therapy is classified as a health supplement rather than a medical treatment in Taiwan. Conducting large-scale trials would require contractual agreements between the principal investigator, material suppliers, and hospitals, as well as an insurance compensation mechanism. The limited number of participants, short usage period, and patient selection constraints may impact the perception of symptom changes in mild COPD cases, while late-stage COPD patients may not exhibit clear therapeutic effects, due to irreversible airway changes.

## 5. Conclusions

Hydrogen therapy holds promise as a potential adjunctive treatment for COPD due to its antioxidant and anti-inflammatory properties. The therapy showed symptom improvements in certain COPD patients. However, in our study, there were no significant differences in inflammation markers, oxidation indices, antioxidant indices, and lung function after one month of usage. Further research is needed to establish the optimal dosing, delivery methods, and long-term effects and to identify specific responder populations. Hydrogen therapy has the potential to revolutionize COPD management and improve outcomes for patients, offering new avenues for therapeutic interventions.

## Figures and Tables

**Table 1:** Basic characteristics of 6 patients with chronic obstructive pulmonary disease, and lung function, 6 MWT, CAT, mMRC, and sleep quality before and after receiving inhaled hydrogen adjuvant therapy.

Inhaled Hydrogen Adjuvant Therapy												
Factors	Patient 1		Patient 2		Patient 3		Patient 4		Patient 5		Patient 6	
Age	77		58		87		65		59		67	
Gender (M/F)	M		F		M		M		M		M	
Smoking status	former		active		former		active		active		never	
Pack-years	20		45		30		30		15		0	
treatment	pre	post	pre	post	pre	post	pre	post	pre	post	pre	post
Post-BD FVC (L)	2.49	2.73	2.59	2.54	1.28	1.36	2.9	2.9	4.5	4.31	1.05	1.01
Post-BD FEV1 (L)	1.11	1.19	1.16	1.17	0.68	0.73	1.55	1.57	2.65	2.62	0.6	0.63
Post-BD FEV1/FVC (%)	44	44	45	46	53	54	53.25	54	59	61	56	63
DLCO (%) mL/mHg/mim/L	52.23	65.53	42.11	34.85	32.9	33.8	17.66	17.37	50.29	50.47	56	66.29
DLCO/VA (%) mL/mHg/mim/L	73.6	79.23	38.26	43.81	96.47	96.45	23.2	21.82	61.18	60.76	118	130.99
CAT	9	9	11	3	25	21	17	11	14	3	18	8
mMRC	2	2	1	0	4	4	4	3	1	0	3	2
Sleep quality	2	2	2	2	1	1	2	2	2	2	1	1
6 MWT Rest SpO_2_%	97	97	98	96	95	96	87	92	98	97	91	92
Lowest SpO_2_%	93	93	92	92	90	92	72	79	92	92	79	87
6 MWD (m)	241	241	425	485	137	152	245.5	137.5	362.5	443.25	463.5	384
MIP (cmH_2_O)	70	70	60	110	80	90	70	70	120	90	60	50
MEP (cmH_2_O)	120	120	90	100	90	90	100	70	80	130	150	120

CAT: changes of Assessment Test; mMRC: modified Medical Research Council Dyspnea Scale; BD: bronchodilator; FVC: forced vital capacity; FEV1: forced expiratory volume in one second; DLCO: Diffusing capacity of the lung for carbon monoxide; VA: alveolar volume; 6 MWT: 6 min walk test; 6 MWD: 6 min walk distance; MIP: maximal inspiratory pressure; MEP: maximal inspiratory pressure; SpO_2_: peripheral capillary oxygen saturation.

**Table 2:** Comparisons of lung function, 6 MWT, CAT, and mMRC of 6 patients with chronic obstructive pulmonary disease before and after receiving inhaled hydrogen adjuvant therapy.

Inhaled Hydrogen Adjuvant Therapy			
Factor	Pre-Treatment Median (IQR)	Post-Treatment Median (IQR)	*p*-Value
Post-BD FVC (L)	2.54 (1.22–3.30)	2.64 (1.27–3.25)	0.893
Post-BD FEV1 (L)	1.14 (0.66–1.83)	1.18 (0.71–1.83)	0.141
FEV1/FVC (%)	53.12 (45–56.75)	54 (45.5–61.5)	0.112
DLCO	46.2 (29.09–53.17)	42.66 (29.69–65.72)	0.345
DLCO/VA	67.39 (34.50–101.85)	69.99 (38.31–105.08)	0.345
CAT	15.5 (10.5–19.75)	8.5 (3–13.5)	0.043
mMRC	2.5 (1–4)	2 (0–3.25)	0.046
Sleep quality	0 (0–1)	0 (0–1)	1.000
Lowest SpO_2_%	91 (77.25–92.25)	92 (85–92.25)	0.109
6 MWD	304 (215–434.63)	312.5 (148.38–452.94)	0.893
MIP (cmH_2_O)	70 (60–90)	80 (65–95)	0.854
MEP (cmH_2_O)	95 (87.5–127.5)	110 (85–122.5)	1.000

CAT: changes of Assessment Test; mMRC: modified Medical Research Council Dyspnea Scale; BD: bronchodilator; FVC: forced vital capacity; FEV1: forced expiratory volume in one second; DLCO: Diffusing capacity of the lung for carbon monoxide; VA: alveolar volume; 6 MWT: 6 min walk test; 6 MWD: 6 min walk distance; MIP: maximal inspiratory pressure; MEP: maximal inspiratory pressure; SpO_2_: peripheral capillary oxygen saturation.

**Table 3:** Blood examination, inflammation markers, and free radicals of 6 patients with chronic obstructive pulmonary disease before and after receiving inhaled hydrogen adjuvant therapy.

Inhaled Hydrogen Adjuvant Therapy			
Factor	Pre-Treatment Median (IQR)	Post-Treatment Median (IQR)	*p*-Value
WBC	6.8 (6.55–7.65)	6.8 (6.05–10.05)	0.500
RBC	4.70 (4.61–4.94)	4.69 (4.50–5.24)	0.892
Hb	14.65 (14.3–15.43)	14.8 (13.9–16.4)	1.000
HCT	43.75 (41.98–48.1)	44.9 (41.83–47.88)	0.498
MCV	92.4 (89.05–96.35)	92.1 (88.85–98.45)	0.892
MCH	31.05 (29.98–32.28)	31.1 (30–32.73)	0.500
MCHC	33.35 (32.83–34.03)	33.7 (32.73–34.33)	0.279
RDW-SD	44.4 (43.4–51.03)	43.9 (42.65–50.25)	0.138
Platelets	247 (209.75–298.75)	260 (232.75–314.5)	0.043
RDW-CV	13.35 (12.5–14.33)	13.1 (12.43–14.1)	0.109
PDW	11.45 (10.53–13.2)	10.65 (10.18–11.48)	0.080
MPV	10.1 (9.75–10.75)	9.85 (9.5–10.25)	0.131
Eosinophil	1.35 (1.03–1.58)	1.95 (1.2–2.95)	0.116
TAC	475 (410–522.25)	513 (432–587)	0.225
GPX	102.6 (85.58–122.7)	104.9 (89.88–121.4)	0.686
MPO	75.1 (55.45–110)	62.05 (48.4–101.9)	0.500
8-OHdG	6.69 (4.09–8.59)	7.32 (3.48–8.38)	0.500
T-CHOL	214 (179.25–241.5)	217.5 (178–263)	0.416
TG	110.5 (69.25–177)	108 (82.75–158.75)	0.686
LDL-C	130.5 (84–165.75)	121 (88–169.25)	0.893
HDL-C	51 (45.75–61.25)	49 (40.75–57.75)	0.136

plasma glutathione peroxidase (GPX); myeloperoxidase (MPO); total antioxidant capacity (TAC), urine 8-hydroxy-2′-deoxyguanosine (8-OHdG).

## Data Availability

The data that support the findings of this study are available from the first author Shih-Feng Liu.

## Abbreviations

COPD, chronic obstructive pulmonary disease; GOLD, Global Initiative for Chronic Obstructive Lung Disease; FEV1, forced expiratory volume during the first second; FVC, forced vital capacity.
